# Book Reviews

**Published:** 1913-08

**Authors:** 


### BOOK REVIEWS

### FIFTEENTH ANNUAL MEETING
of the
### AMERICAN PROCTOLOGIC SOCIETY

HELD AT MINNEAPOLIS, MINN., JUNE 16 AND 17, 1913

The President, Dr. Louis J. Hirschman, of Detroit, Mich., in the chair.

OFFICERS ELECTED FOR THE ENSUING YEAR.

President, JOSEPH M. MATHEWS, M. D.,

Louisville, Ky.

Vice-President, JAS. A. MAC MILLEN, M. D.,
Detroit, Mich.

Secretary-Treasurer, ALFRED J. ZOBEL, M. D.,
San Francisco, Cal.

EXECUTIVE COUNCIL.

LOUIS J. HIRSCHMAN, M. D., Detroit, Mich.
J. RAWSON PENNINGTON, M. D., Chicago, Ill.
WM. M. BEACH, M. D., Pittsburgh, Pa.
ALFRED J. ZOBEL, M. D., San Francisco, Cal.

The following were elected Associate Fellows of the Society:

V. Lee Fitzgerald, 17 Batley St., Providence, R. I.

J. M. Frankenburger, Rialto Bldg., Kansas City, Mo.

Wm. H. Kiger, 308 Consolidated Bank Bldg., Los Angeles, Cal.

Walter I. Le Fevre, 218 Lennox Bldg., Cleveland, Ohio.

---

The following is an abstract of the principal papers read:

### PRESIDENT'S ADDRESS—PROCTOLOGY AND PROCTO-ENTEROLOGY.

BY LOUIS J. HIRSCHMAN, OF DETROIT, MICH.

He stated that, "Proctology come into its own," is in reality the study of the entire intestinal tract its diseases and their remedies. A Proctologist becomes skilled to a high degree in the medicinal and surgical treatment of the diseases of the lower bowel. A medical practitioner, sufficiently skilled and competent to treat diseases affecting any portion of the intestinal tract, should be competent to treat all portions. The modern Proctologist, therefore, must be an intestinal surgeon. He must have some knowledg of modern views and discoveries bearing on the digestive tract, as they have a direct bearing on intestinal function and pathology. He should no more limit his activities to the rectum and sigmoid alone than does the laryngologist to the larynx, or the unrologist to the urethra.

An arbitrary line of division which limits a specialist's activities to the lower six or eight inches of the colon is absurd. The Proctologist has no moral right to withhold his special skill in intestinal surgery from the patient who suffers from diseases of the small intestine or upper colon. The largq problems of intestinal stasis, chronic inflammatory conditions, and malignant diseases of the small and large intestines, demand the best that is in every Fellow of our organization. He should ever study and fathom out the problems of etiology, pathology, and proper therapy.

The establishment of a section on Gastro-Enterology and Proctology in the American Medical Association would greatly increase the value of that organization to every one of its members who comes in contact with diseases of the alimentary tract.

It is the American Medical Association which should foster all that is new and valuable in medicine. It is the greatest medical educational institution in our country; and the Fellows of the American Proctologic Society should be the most enthusiastic supporters of such a section, if established.

---

### MEMOIR OF JAMES P. TUTTLE, New York City, N. Y.
### and
### MEMOIR OF LOUIS STRAUS, St. Louis, Mo.

By Joseph M. Mathews, M. D., of Louisville, Ky.

These memoirs were inspired by precious memories of close personal association with the late Fellows of the American Proctologic Society, who were both character members of that organization. In well chosen and deeply sympathetic words the noble character and high professional worth of these lamented Fellows were outlined in a manner which did honor to their memory.

### A REVIEW OF PROCTOLOGIC LITERATURE FROM MARCH, 1912, TO MARCH, 1913.

By Samuel T. Earle, M. D., of Baltimore, Md.,
Chairman of the Committee on the Same.

In this review of Proctologic literature, Earle quotes freely from the following authors:—

Dr. Edward H. Goodman, of Philadelphia, Pa. (Progressive Medicine, December, 1912, page 100), quoting from Ageron, (Archix f. Verdauungskrankheiten, 1911, XVII, page 584), "Constipation."

W. Ernest Miles, F. R. C. S., England, (The Glascow Medical Journal, No. 11, February, 1912, page 82), "The Treatment of Carcinoma of the Rectum and Pelvic Colon."

H. Graeme Anderson, M. B., Ch. B., F. R. C. S., (British Medical Journal, 1912, Vol. I, page 129), "Solid Carbon Dioxide in the Treatment of Hemorrhoids."

Dr. Walton Martin, New York City, Vol. LV, 1912, page 901), "Carcinoma of the Rectum: Combined Abdominal and Perineal Rectectomy."

Harrison Cripps, F. R. C. S., England, (British Medical Journal, 1912, Vol. 2, page 843), "The Treatment of Rectal Cancer."

Dr. William J. Mayo, Rochester, Minn., (Annals of Surgery, Vol. 56, 1912, page 240), "The Radical Operation for the Relief of Cancer of the Rectum and Rectosigmoid."

Mr. Lockhart Mummery, (British Medical Journal, Vol. 1, 1912, page 1427), "Recorded Cases of Intractable Constipation Treated by Operation."

Dr. Arthur W. Elting, Albany, New York, (Transactions of the American Surgical Association, 1912, Vol. XXX, page 176), "Treatment of Fistulo in Ano, with Special Reference to the Whitehead Operation."

Dr. Alexis V. Moschowitz, New York City, (New York State Journal of Medicine, Vol. XII, No. 11, page 654), "The Pathogenesis, Anatomy and Cure of Prolapse of the Rectum."

Dr. William C. Lusk, of New York City, (Annals of Surgery, January, 1913, Vol. LVII, No. 1, page 106), gives a description of, "An Instrument for Establishing Fecal Drainage, with a Report of its use on a Case, and a Consideration of the Site for Making a Fecal Fistula in Low-seated Intestinal Obstruction."

Two new proctoscopes have been devised during the past year. "The 1912 Proctoscope," Jerome M. Lynch, (New York Medical Journal, 1912).

"A new Pneumo-electric proctoscope and sigmoidoscope," F. C. Yeoman, (Journal of American Medical Association, 1912, Vol. LVIII, page 929).

There have been several reports of the use of Extra-dural Sacral Anaesthesia, von W. Stockel, (Zentralblatt fur Gynakologie, No. 1, 1909), von Dr. Maryan Tobiaszek, (Zentralblatt fur Gynakologie, 33 Jahgang, 1909), and Dr. Jerome M. Lynch, (Medical Record, February 8, 1913, page 235).

---

A Method of Operating on Fistula without Cutting Muscular Tissue. By Rollin H. Barnes, M. D., of St. Louis, Mo.

This method is used in those cases of fistulae which involve the sphincter muscles. An incision is made external to the sphincter, similar to that made when incising an ischio-rectal abscess. Through this opening the scar tissue is dissected out up to the internal opening. An incision is then made at the skin margin, so that the middle of this incision passes through an imaginary longitudinal line drawn from the internal opening. Gauze drainage is kept in this until the external wound is healed sufficiently. Then the submucous tract, which remains, is incised under local anaesthesia. No muscular tissue having been cut, the function of the sphincters is preserved intact.

(To be continued.)

---

In determining between malaria and pyogenic infection as the cause of a chill and pyrexia a leucocytoiss of 20,000 or less roes not necessarily exclude paludism; it is sometimes found at the outset of a malarial attack.

BLOOD PRESSURE. From the Clinical Standpoint. By Francis Ashley Faught, M. D., of the Medico-Chirurgical College, Philadelphia. Octavo of 281 pages, illustrated. Philadelphia and London: W. B. Saunders Company, 1913. Price $3.00 net.

The clinical value of the sypygmomanometer is no longer denied. Faugh has attractively presented the pith of medical literature bearing on blood pressure studies in their relation to medicine, not only in cardio-vascular and rena lconditions, but also in many diseases in which clinical observation has shown information obtained by the sphygomomanometer to be of value.

---

PROGRESSIVE MEDICINE. A Quarterly Digest of Advances, Discoveries and Improvements in the Medical and Surgical Sciences. Edited by H. A. Hare, M. D. Assisted by L. F. Appleman, M. D. Vol. II., June, 1913. Lea & Febiger, Philadelphia.

The subject of Hernia is reviewed by Coley; Surgery of the Abdomen, exclusive of hernia, by Gerster; Gynecology by Clark; Blood, etc., by Stengel, and Ophthalmology by Jackson.

---

THE SURGICAL CLINICS OF JOHN B. MURPHY, M. D., at Mercy Hospital, Chicago. Volume II. Number III. (June 1913.) Octavo of 185 pages, 62 illustrations. Philadelphia and London: W. B. Saunders Company, 1913. Published Bi-Monthly. Price per year; Paper, $8.00. Cloth, $12.00.

The present volume of the "Surgical Clinics of John B. Murphy" is well up to the usual standard of excellence.

---

COLLECTED PAPERS BY THE STAFF OF ST. MARY'S HOSPITAL (MAYO CLINIC) FOR 1912. Octavo of 942 pages, 219 illustrations. Philadelphia and London: W. B. Saunders Company, 1913. Cloth, $5.50 net.

This is the sixth volume of the collected papers of the Mayo staff and cointains the articles written and presented for publication to the various Medical Journals during the year 1912.

